# Frequency of helping friends and helping strangers is explained by different neural signatures

**DOI:** 10.3758/s13415-018-00655-2

**Published:** 2018-11-07

**Authors:** Anne Saulin, Thomas Baumgartner, Lorena R. R. Gianotti, Wilhelm Hofmann, Daria Knoch

**Affiliations:** 1 Institute of Psychology, Department of Social Psychology and Social Neuroscience, University of Bern, Fabrikstrasse 8, 3012 Bern, Switzerland; 2 Social Cognition Center, University of Cologne, Cologne, Germany

**Keywords:** Experience sampling, Neural signature, Daily helping, Helping strangers, Helping friends, Resting EEG, LORETA

## Abstract

Acts of helping friends and strangers are part of everyday life. However, people vary significantly with respect to how often they help others and with respect to whom they actually help on a day-to-day basis. Despite everyday helping being so pervasive, these individual differences are poorly understood. Here, we used source-localized resting electroencephalography to measure objective and stable individual differences in neural baseline activation in combination with an ecologically valid method that allows assessment of helping behavior in the field. Results revealed that neural baseline activation in the right dorsolateral prefrontal cortex (DLPFC) – a brain region associated with self-control and strategic social behavior – predicts the daily frequency of helping friends, whereas the daily frequency of helping strangers was predicted by neural baseline activation in the dorsomedial prefrontal cortex (DMPFC) – a brain region associated with social cognition processes. These findings offer evidence that distinct neural signatures and associated psychological and cognitive processes may underlie the propensity to help friends and strangers in daily life.

**Electronic supplementary material:**

The online version of this article (10.3758/s13415-018-00655-2) contains supplementary material, which is available to authorized users.

## Introduction

Helping other people is ubiquitous in our daily life. However, people vary considerably in the extent to which they help others (Amato, 1983, 1990; Hein, Silani, Preuschoff, Batson, & Singer, 2010; Severy, 1975), as well as with respect to whom they actually help (Amato, 1990; Nielson, Padilla-Walker, & Holmes, 2017; Rameson, Morelli, & Lieberman, 2012). Early works by Amato (1990) demonstrated differences between daily episodes of helping friends compared to daily episodes of helping strangers. Specifically, people varied considerably in the daily frequency of helping friends and strangers and reported that helping friends tended to be planned, whereas helping strangers tended to be more spontaneous in nature. Both kinds of helping behavior are important to uphold cooperation on the level of peer connections as well as on a societal level at large (Alexander, 1987; Lenrow, 1978; Nowak, 2006; Padilla-Walker, Carlo, & Nielson, 2015).

This variability in helping behavior raises the following questions: What causes these individual differences in helping behavior? Are the same sources driving the individual differences in helping behavior towards friends and strangers or are different sources responsible for the variability in helping friends and strangers? Previous studies that tried to understand these individual differences in helping behavior mostly relied on hypothetical helping scenarios and used self-report questionnaires as personality measures (e.g., empathic concern, Maner & Gailliot, 2007; agreeableness, Graziano, Habashi, Sheese, & Tobin, 2007; self-regulation, Padilla-Walker & Christensen, 2011). These studies did a good job in differentiating psychological processes of helping friends and helping strangers. The present study tries to uncover the sources of individual differences in these two forms of helping behavior by using a neurobiological approach. We used a neural trait measure (e.g., Nash, Gianotti, & Knoch, 2015) in combination with a measure of interpersonal help.

An ideal neural trait measure to uncover neural sources is task-independent baseline activation measured by resting electroencephalography (EEG) because this measurement demonstrates high stability over time and high specificity (i.e., the extent to which an EEG pattern uniquely belongs to a given person). Studies investigating the stability of resting EEG revealed test-retest reliabilities of up to 0.8 over a period of up to 5 years (Dünki, Schmid, & Stassen, 2000; Näpflin, Wildi, & Sarnthein, 2007; Smit, Posthuma, Boomsma, & De Geus, 2005) and studies exploring the specificity revealed recognition rates of up to 99% (Dünki et al., 2000; Näpflin et al., 2007). Due to high intra-individual stability and specificity, this measurement provides an ideal neural trait marker to investigate the individual differences in friend-helping and stranger-helping behavior. Further, prior literature linking psychological processes to neural functioning shows that neural signatures allow inferences about the psychological and cognitive processes that underlie differences in behavior (e.g., Baumgartner, Gianotti, & Knoch, 2013; Boersma et al., 2011; Nakao, Bai, Nashiwa, & Northoff, 2013; Schiller, Gianotti, Nash, & Knoch, 2014). Thus, neural signatures associated with certain functions can provide evidence for the underlying processes that may impact people’s helping behavior.

To measure behavioral variation in helping behavior towards friends and strangers in an ecologically valid way, we used the experience sampling method, a method that allows for prompt ecological assessment of behavior in the participants’ daily life (Csikszentmihalyi & Larson, 2014; Hofmann & Patel, 2015; Hofmann, Wisneski, Brandt, & Skitka, 2014; Shiffman, Stone, & Hufford, 2008). This method enables us to capture participants’ helping behavior and experiences as they occur in people’s natural environments. For this purpose, participants were “pinged” on their smartphone multiple times a day and asked to immediately complete a short survey tapping into behavior that had occurred recently. Because participants only have to remember actions within the last hour(s), and data are collected over multiple days, this method makes it easier for participants to accurately recall behaviors (Scollon, Chu, & Diener, 2003).

Previous neural studies on helping behavior focussed on “online” (i.e., task-dependent) measures such as brain activity during a helping paradigm or an empathy task, and all but one of these studies (see Rameson et al., 2012 for an exception) did not differentiate helping behavior towards friends and strangers. These studies found that task-dependent activity in brain areas associated with mentalizing (e.g., DMPFC, temporo-parietal junction), empathy (e.g., insula), and self-control (e.g., DLPFC) was positively associated with helping behavior (FeldmanHall, Dalgleish, Evans, & Mobbs, 2015; Hein et al., 2010; Hu, Strang, & Weber, 2015; Masten, Morelli, & Eisenberger, 2011; Telzer, Masten, Berkman, Lieberman, & Fuligni, 2011; Tusche, Bockler, Kanske, Trautwein, & Singer, 2016; Waytz, Zaki, & Mitchell, 2012) (for review see Chakroff & Young, 2014). Such studies thus do not identify stable neural traits that may explain a person’s inclination to help friends or help strangers. However, these studies may provide a tentative hint about potential neural traits underlying these differences. In this context, it is important to note that the present study is of an exploratory nature and that the previous literature does not allow for clearly derivable hypotheses. Hence, we conducted exploratory whole-brain corrected analyses to uncover the neural sources of differences in friend-helping and stranger-helping behavior.

## Methods

### Participants

Eighty-seven healthy students from the University of Bern (21 male, mean age: 20.9 ± 2.0 years) took part in the experiment. We recruited participants for one academic semester and collected as much data as possible during that time. Data were analyzed after the testing was complete.

This study was approved by the local ethics committee of the Faculty of Human Sciences (University of Bern, Switzerland). All subjects gave written informed consent in accordance with the Declaration of Helsinki, and were informed of their right to discontinue participation at any time. Participants received a monetary compensation of 25 Swiss francs (CHF; 1 CHF ≈ 1 US$) and an additional 2 CHF for each completed daily response, resulting in a maximum possible amount of 55 CHF.

### Procedure

In an initial session, resting EEG data were obtained (for details see below). After an interval of several weeks (mean = 22.9, SD = 10.4), participants came back to the lab to be familiarized with the use of the experience sampling method and the content of the items (for details see below). On this occasion, participants were also registered with the online software that distributed the links to the survey via text message (SurveySignal). At the end of this second session, participants filled out several personality questionnaires (for details see below). Two days after this laboratory session, the actual experience sampling started. The daily assessment of helping behavior took place during the semester, so that participants had plenty of opportunities to experience situations in which they could be helpful.

#### Daily assessment of helping behavior

The daily helping survey was implemented using the Qualtrics survey software (“Qualtrics”, 2005). The link to the online survey, which was optimized for use on smartphones, was distributed via text message using the online service SurveySignal (Hofmann & Patel, 2015). Participants received the link to the daily helping behavior survey three times per day (late morning, afternoon, evening) for 5 consecutive days. After these 5 days, participants were informed about their final compensation and were paid. On average, participants responded at 14.8 of the 15 experience sampling time points (SD: 0.69; range: 12–15).

The daily helping survey consisted of 13 daily helping behaviors (e.g., “I offered my seat to someone on the bus/tram” or “I let someone in front of me in the queue at the supermarket”; see Online Supplementary Material for a list of all items used in the daily survey) inspired by Morrelli and colleagues (Morelli, Rameson, & Lieberman, 2014). In addition, participants were able to manually add rare types of helping behavior, such as helping a friend to move house. For each behavior, participants were asked to indicate whether they had shown this behavior since last answering the survey, and if so, who the beneficiary of this behavior was. That is, they should indicate whether they helped a friend or a stranger. Helping family members was explicitly not included, since it has been shown that helping family is different from helping non-family members (Amato, 1983). Additionally, participants were able to indicate at the end of each survey whether there were particular circumstances that influenced their opportunities to help (e.g., they were sick at home thus not being able to experience situations in which they could be helpful). None of our subjects reported such circumstances.

#### Personality questionnaires

In order to examine whether individual differences in neural baseline activation explain unique variance in helping behavior compared to subjective trait measures, participants answered the following well-established personality questionnaires. The prosocial tendencies measure (PTM, Carlo & Randall, 2002), which encompasses six subscales, was used. Each subscale refers to the nature of the circumstances under which the participant helps most preferably: public (e.g., “Helping others when I am in the spotlight is when I work best”), anonymous (e.g., “I think that helping others without them knowing is the best type of situation”), dire (e.g., “It is easy for me to help others when they are in a dire situation”), emotional (e.g., “I tend to help others particularly when they are emotionally distressed”), compliant (e.g., “I never hesitate to help others when they ask for it”), and altruism (e.g., “One of the best things about charity is that it looks good on my resume”). Further, the empathic concern and the perspective-taking subscales of the interpersonal reactivity index (IRI, Davis, 1983) were measured. Due to technical problems, personality questionnaire data by three participants were not saved, resulting in a total of 84 sets of questionnaire data.

#### Recording and processing of the EEG data

A continuous EEG was recorded from 64 Ag/AgCl electrodes arranged in the 10–10 system montage (Nuwer et al., 1998) with a sampling rate of 500 Hz (bandwidth: 0.1–250 Hz). Electrode FCz was used as the recording reference and CPz as the ground electrode. Horizontal and vertical electro-oculographic signals were recorded with two additional electrodes at the left and right outer canthi of the left and right eye and an additional electrode at the left infraorbital. Impedances were kept below 10 kΩ. Participants were seated in a sound and electrically shielded chamber that was dimly lit and contained an intercom connection to the experimenters. EEG was recorded during rest with open or closed eyes; the instructions about eye opening/closing were given via intercom. The protocol consisted of 20-s eyes open followed by 40-s eyes closed, repeated five times (such a protocol guarantees minimal fluctuations in participants’ vigilance state). In line with many previous neural trait studies (e.g., Baumgartner et al., 2013; Gianotti et al., 2009; Kam, Bolbecker, O’Donnell, Hetrick, & Brenner, 2013; Li et al., 2017; Vecchio et al., 2013), data analysis is based on the 200-s eyes-closed condition (with the exception of Online Supplementary Analyses 3) because there is no visual input in this condition, and therefore the eyes-closed condition is closer to a resting state than the eyes-open condition.

For preprocessing, EEG data were filtered using a high-pass filter of 0.5 Hz and a low-pass filter of 30 Hz, notch filter enabled at 50 Hz. EEG signals with excessive noise were replaced using a spline interpolation of the signal of adjacent electrodes. To remove eye movements, we first ran an Independent Component Analysis (ICA) and then manually removed factors related to horizontal and vertical eye movements (usually only two factors were detected and eliminated). After having removed the factors related to eye movements, the EEG was recomputed using an inverse ICA procedure. Then we applied an automatic artifact rejection with the following parameters: maximal voltage step: 15 μV; maximal amplitude: ± 100 μV; minimal allowed activity in intervals of 100-ms length: 0.5 μV. After this automatic artifact rejection, data were also visually examined to eliminate residual artifacts. Further, data were recomputed against the average reference and re-sampled to a power of 2 (512 Hz). All artefact-free 2-s epochs of the eyes-closed condition were extracted through a Hanning window (window length 10%). There were on average 88 epochs per person. After applying a Fast Fourier Transformation to calculate power spectra, with a 0.5-Hz resolution, the spectra of each participant were averaged for each channel over all epochs. Absolute power values were obtained for the following seven independent frequency bands (Herrmann, Fichte, Freund, et al., 1979): delta (1.5–6 Hz), theta (6.5–8 Hz), alpha1 (8.5–10 Hz), alpha2 (10.5–12 Hz), beta1 (12.5–18 Hz), beta2 (18.5–21 Hz), and beta3 (21.5–30 Hz).

#### Intracortical source localization

Standardized low-resolution brain electromagnetic tomography (sLORETA, Pascual-Marqui, 2002) was used to calculate the intracortical electrical sources that generated the scalp-recorded activity in each of the seven frequency bands described above. sLORETA applies a distributed source localization technique that approaches the inverse problem without an *a priori* assumption regarding the number of underlying sources and computes electric neural activity as standardized current density (unit: amperes per square meter, A/m^2^). The sLORETA solution space consisted of 6,239 voxels (voxel size: 5 mm × 5 mm × 5 mm) and was restricted to cortical gray matter, as defined by the digitized Montreal Neurological Institute (MNI) probability atlas. The sLORETA technique has been validated by various studies using a variety of methods, such as combined sLORETA and fMRI or Positron Emission Topography (PET) studies as well as studies employing experimental paradigms for which the neural generators are well known (Laxton et al., 2010; Olbrich et al., 2009; Zumsteg, Lozano, Wieser, & Wennberg, 2006). In order to reduce confounds that have no regional specificity, such as total power inter-subject variability, a global subject-wise normalization of the whole-brain sLORETA images was carried out. This normalization procedure scales all the brain data of one subject with the grand-average over all frequency bands and voxels, and thereby eliminates variability due to factors such as variable scalp and skull conductivity that have no regional specificity. For statistical analyses, the sLORETA images were log-transformed.

#### Behavioral and psychometrical scores

Based on the data obtained in the daily online survey, mean daily helping scores were computed. Scores of helping behavior were computed by summing the number of helping acts and dividing by the number of days participated. This was done for each recipient category separately resulting in one score for mean daily friend-helping behavior and one for mean daily stranger-helping behavior. In order to check whether participants’ helping behavior changed over the 5 days or whether it is rather stable, we conducted a repeated-measures ANOVA with day (1–5) as the time variable and either daily friend-helping behavior or daily stranger-helping behavior as the dependent variable. We found no significant differences between the 5 days, neither for friend-helping behavior (F (4, 82) = 0.308, p = 0.819) nor for stranger-helping behavior (F (4,82) = 0.552, p = 0.648). Thus, these findings suggest that the mean daily helping scores reflect stable individual differences in helping behavior.

### Statistical analyses

Analyses focused on the correlation between behavioral and brain data. Regression analyses were performed, correlating the behavioral measures of interest (mean daily friend-helping and mean daily stranger-helping behavior) with the log-transformed sLORETA images in each frequency band. To incorporate correction for multiple testing, a nonparametric approach was employed. This approach uses a randomization strategy (Nichols & Holmes, 2001) that determines the values of the critical probability threshold for the observed r-values in order to identify cortical voxels that significantly correlate with the measures of interest. In a next step, for regions that displayed significant, whole-brain-corrected correlations with the measures of interest, the respective voxel with the strongest correlation (greatest r-value) was used as the center for spherical regions of interest (ROIs; radius: 10 mm). Averaged current density values were then extracted for all voxels within these ROIs for visualization and further analyses.

In an additional step, robust regressions were carried out with the extracted values using R statistics software (R Core Team, 2014). In these analyses, mean current density values of the respective ROIs were used as predictors and mean daily friend-helping and mean daily stranger-helping behavior as the respective dependent variables. These additional ROI-based robust regression analyses were conducted to minimize the influence of potential outliers, which cannot be done within LORETA. The data were extracted from LORETA for these analyses as well as for the corresponding scatterplots in order to further corroborate and visualize the association between neural data and daily helping behavior.

## Results

On average, participants reported 8.7 ± 4.4 (mean ± SD) acts of helping per day. Among those, there were 7.3 ± 3.8 (mean ± SD) acts of helping friends and 1.4 ± 0.9 (mean ± SD) acts of helping strangers per day. Independent t-tests showed no gender effects in the frequency of helping friends (t = 1.36, p = 0.18) and helping strangers (t = -0.62, p = 0.54). Interestingly, mean daily friend-helping and mean daily stranger-helping behavior was not correlated (r = 0.02, p = 0.89). This indicates that different neural sources might explain individual differences in helping behavior towards friends and/or strangers.

**Fig. 1:**
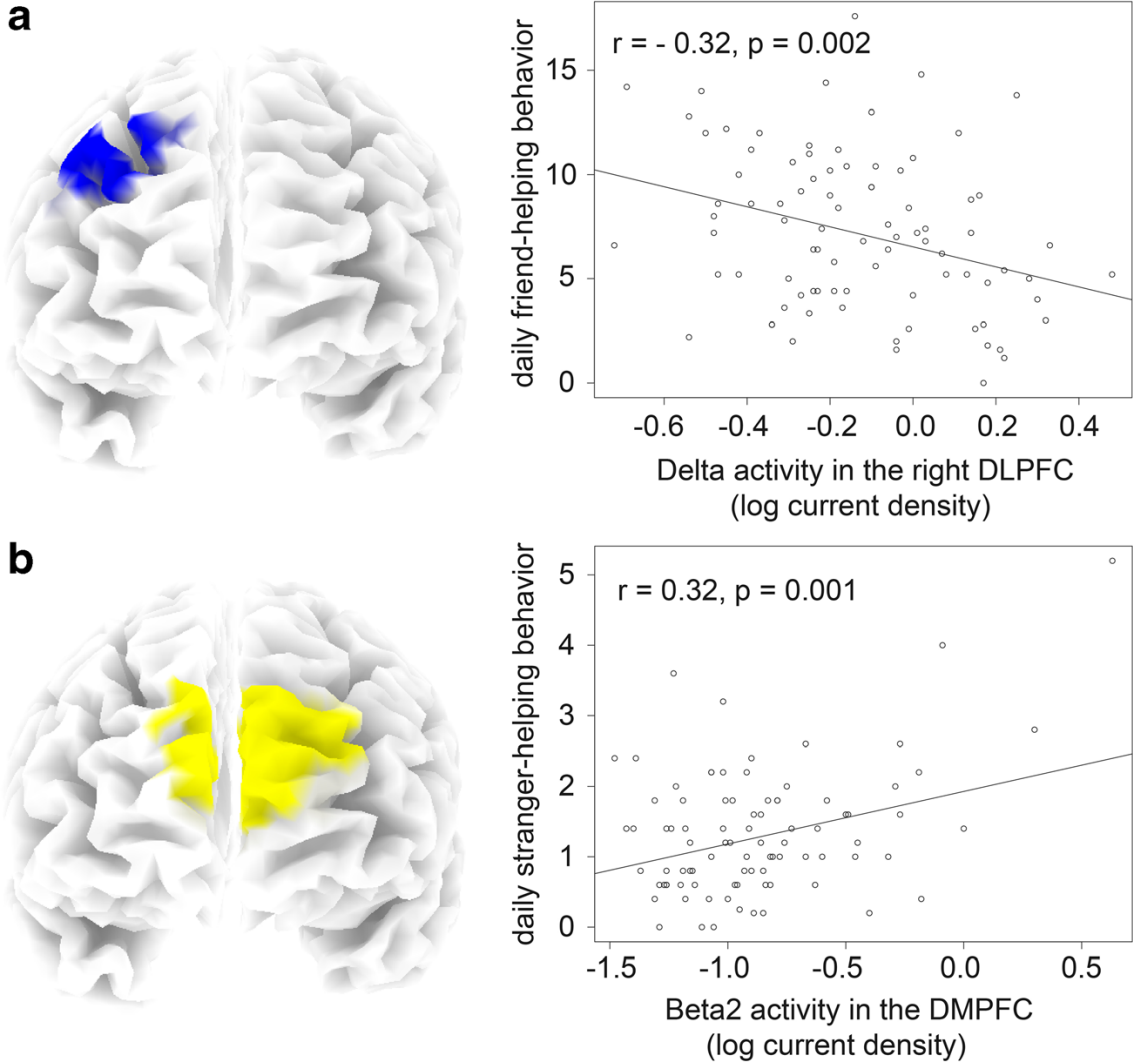
Visualization of the whole-brain-corrected significant correlations (p < 0.05, corrected) between (**A**) mean daily friend-helping behavior and neural baseline activation in the delta frequency band in right dorsolateral prefrontal cortex (DLPFC), and (**B**) mean daily stranger-helping behavior and neural baseline activation in the beta2 frequency band in dorsomedial prefrontal cortex (DMPFC). The right panel comprises the corresponding scatterplots showing regression lines and r-values, which are re-computed by robust regressions to reduce the effect of potential outliers. The scatterplots are based on the extracted values from 10-mm spheres around the corresponding MNI peak coordinates

Whole-brain corrected analyses revealed a significant negative correlation between mean daily friend-helping behavior and current density in right DLPFC (MNI peak coordinates: x = 35, y = 45, z = 35) in the delta frequency band. This correlation comprised a total of 34 voxels (voxelsize: 5 mm^3^) at p < .05 (whole-brain corrected). Robust regression using the mean current density in a ROI (10-mm sphere around the peak voxel) resulted in a negative correlation coefficient of -0.32 (p = 0.002; see Fig. 1A). Partialling out participants’ gender and age did not affect the results (r = -0.33, p = 0.001). Since resting slow-wave oscillations in the delta band likely reflect decreased cortical activation (e.g., Modarres, Kuzma, Kretzmer, Pack, & Lim, 2017; Pizzagalli et al., 2004; Riedner, Hulse, Murphy, Ferrarelli, & Tononi, 2011), our results indicate that the higher a person’s baseline activation in the right DLPFC, the more that person helps friends in daily life. No other regions or frequency bands yielded significant correlations with mean daily friend-helping behavior nor did mean daily stranger-helping behavior correlate with baseline activation in the right DLPFC at a whole-brain-corrected significance threshold. Even an ROI-based post hoc test in the DLPFC revealed no such association in the delta band for daily stranger-helping behavior (r = 0.13, p = 0.209). Finally, controlling for mean daily stranger-helping behavior in the regression analysis did not significantly attenuate the association between mean daily friend-helping behavior and baseline activation in the right DLPFC (r = -0.33 p < 0.001, current density in the delta band), suggesting that this association is highly specific.

In contrast, mean daily stranger-helping behavior was positively correlated (whole-brain corrected) with current density in the DMPFC in all three beta frequency bands (beta1, 254 voxels; beta2, 178 voxels; and beta3, 197 voxels; same MNI peak coordinates in all three beta bands: x = -5, y = 65, z = 20) and the alpha2 frequency band (162 voxels, MNI peak coordinates: x = -15, y = 65, z = -10). Robust regression analyses were conducted on the extracted values from a ROI (10-mm sphere around the peak voxel) in the significant frequency bands. These analyses revealed positive correlation coefficients of 0.30 for beta1 (p = 0.001), 0.32 for beta2 (p < 0.001), 0.30 for beta3 (p = 0.002), and 0.27 for alpha2 (p = 0.004). For an illustration of these findings, please see Fig. 1B (in which we depicted the strongest finding in the beta2 band) and Online Supplementary Fig. 1 (for all the other findings). Partialling out participants’ gender and age did not affect the results (beta1: r = 0.31, p = 0.001; beta2: r = 0.33, p < 0.001; beta3: r = 0.31, p = 0.001; alpha2: r = 0.28, p = 0.004). Since resting fast-wave oscillations likely reflect increased cortical activation (e.g., Laufs et al., 2003; Oakes et al., 2004), these findings indicate that the higher a person’s baseline activation in the right DMPFC, the more this person helps strangers in daily life. No other regions or frequency bands yielded significant correlations with mean daily stranger-helping behavior nor did mean daily friend-helping behavior correlate with baseline activation in the DMPFC at a whole-brain-corrected significance threshold. Even an ROI-based post hoc test in the DMPFC revealed no such association in the beta1 (r = -0.07, p = 0.522), beta2 (r = -0.09, p = 0.423), beta3 (r = -0.12, p = 0.266), and alpha2 band (r = -0.08, p = 0.459) for daily friend-helping behavior. Finally, controlling for mean daily friend-helping behavior in the regression analyses did not significantly attenuate the association between mean daily stranger-helping behavior and baseline activation in the DMPFC (r = 0.33, p < 0.001, here we report the finding of the beta2 band only, but the findings hold for the other bands), suggesting that this association is highly specific.

In a final step, we sought to examine whether baseline activation in the DLPFC and the DMPFC explain unique variance in helping friends and strangers that personality questionnaires cannot explain. To this end, we employed eight scales of two well-established personality questionnaires related to helping behavior and associated psychological processes, such as perspective-taking and empathy (six scales of the prosocial tendencies measures – Carlo & Randall, 2002, and two scales of the interpersonal reactivity index – Davis, 1983). We then conducted a model comparison for nested robust regression models. The first model included all eight scales of the personality questionnaires, whereas the second model comprised those same scales as well as the respective brain data as predictors (for simple correlations between helping behavior and scales, please see Online Supplementary Analyses 1).

Findings revealed that both neural signature measurements (baseline activation in the DLPFC and DMPFC) are capable of explaining unique variance in helping behavior, which cannot be explained by the eight scales. More precisely, the eight scales and the baseline activation in the right DLPFC were able to explain 24% of the individual variance in daily friend-helping behavior. However, about a third of this variance, namely 9%, could be accounted for solely by baseline activation in the DLPFC (**Δ**R^2^ = 0.09, p = 0.016, for details see Online Supplementary Material, Table 2/3). A similar picture emerged for daily stranger-helping behavior. All measurements together (all eight scales and the baseline activation in the DMPFC) were able to explain 27% of individual variance in helping strangers. However, almost half this variance, namely 12%, could be accounted for solely by baseline activation in the DMPFC (**Δ**R^2^ = 0.12, p = 0.001, for details see Online Supplementary Material, Table 2/4; here we report the finding of the beta2 band only, but a highly similar picture emerges for the other fast bands). Notably, these findings also hold when controlling for the demographic variables age and gender (for details see Online Supplementary Material, Tables 5, 6, and 7).

As suggested by anonymous reviewers, we conducted several additional analyses to further corroborate our findings. More precisely, we examined whether the resting EEG is stable over time in our sample (see Online Supplementary Analyses 2), whether we find a similar predictive pattern in the eyes-open condition as we have demonstrated for the eyes-closed condition in the results section (see Online Supplementary Analyses 3), and whether the differences in the delay between the acquisition of the resting EEG and the experience sampling affect our results (see Online Supplementary Analyses 4). Notably, all additional analyses further corroborated the main findings reported in the paper, i.e. we found evidence that the resting EEG is highly stable over time in our sample, that the resting EEG in the eyes-open condition shows a highly similar predictive pattern in the DLPFC and DMPFC (as the resting EEG in the eyes-closed condition), and that the differences in the delay between the acquisition of our measurements did not have an impact on our results.

## Discussion

We adopted a neural trait approach in combination with the measurements of field behavior in order to gain an improved understanding of the differences in helping behavior towards friends and strangers in daily life. In line with previous behavioral research (Amato, 1983; Padilla-Walker et al., 2015), we found that daily friend-helping behavior and daily stranger-helping behavior was not correlated on a behavioral level, suggesting that distinct neural signatures might drive the differences in friend-helping and stranger-helping behavior. Indeed, our study revealed such distinct neural signatures for helping friends and strangers. More precisely, while the frequency of daily friend-helping behavior was associated with neural baseline activation in the right DLPFC, the frequency of daily stranger-helping behavior was associated with baseline activation in the DMPFC. Moreover, these neural signatures were able to explain substantial unique variance in helping behavior towards friends and strangers that could not be explained by various self-report measures alone.

The frequency of friend-helping behavior was positively associated with neural baseline activation in the DLPFC. In order to try to interpret this brain finding one might begin by thinking about what differentiates helping a friend compared to helping a stranger on a behavioral level. Both helping a friend and helping a stranger often carry a cost (such as time and physical or psychological effort; Steinbeis, 2018; Lanaj et al., 2016). However, helping a friend comprises additional elements. Namely, there are potential benefits from being prosocial, both in the short- and in the long-term, due to a high possibility of reciprocity: I help you, you help me. Without such an overall sense of “tit-for-tat,” a friendship is unlikely to persist over a long period of time. Thus, maintaining a friendship might require, amongst other elements, a strategic thinking process/capacity that accommodates this aspect of a friendship. In line with this argumentation is an early observation by Amato (1990). He discovered that in contrast to helping strangers, helping friends is most often planned rather than spontaneous. As such, it is reasonable to assume that the planned helping act toward a friend may require increased capacity for strategic social behavior compared to helping a stranger more spontaneously. Further, such a strategic view on helping has been prominently formulated in social exchange theories. They state that helping can be interpreted as a service that can be exchanged between humans (e.g., Blau, 1964; Emerson, 1976; Thibaut & Kelley, 1978). That is, when you help someone, you expect something in return. Of course, such an expectation can only be fulfilled if you see this person again in the future. This criterion holds for daily friend helping but not stranger helping, implying a strategic aspect of friend helping. There is strong evidence that the DLPFC is involved in strategic decision making (e.g., Gianotti, Nash, Baumgartner, Dahinden, & Knoch, 2018; Ruff, Ugazio, & Fehr, 2013; Soutschek, Sauter, & Schubert, 2015; Spitzer, Fischbacher, Herrnberger, Grön, & Fehr, 2007; Strang et al., 2015). Also, developmental neuroscience studies provided insights into the role of self-control and underlying functions of the DLPFC in strategic decision making (e.g., Crone & Steinbeis, 2017; Decety & Cowell, 2016; Steinbeis, Bernhardt, & Singer, 2012). For example, Steinbeis et al. (2012) showed an increase in self-control and strategic behavior with age, which was associated with functional and structural differences of the DLPFC. This finding suggests that the functional role of the DLPFC in strategic decision-making may involve aspects of self-control, i.e., sacrificing for example money or time. Combining the findings of these studies with our current results leads us to speculate that the level of baseline activation in the right DLPFC might reflect a degree of self-control capacity to implement socially smart strategies of behavior, ensuring sustained goodwill for present and future interactions with friends. This capacity may lead to more frequent friend-helping behavior in everyday life. However, please note that our study is only a first step and does not allow narrowing down and disentangling the underlying processes driving friend-helping behavior in the DLPFC precisely.

In contrast to friend-helping behavior, the frequency of stranger-helping behavior was positively associated with neural baseline activation in the DMPFC. This area has consistently been observed to be involved in social cognition processes, such as mentalizing and perspective-taking (e.g., Denny, Kober, Wager, & Ochsner, 2012; Lieberman, 2007; Van Overwalle, 2009). For example, people who appear to be more skilled in putting themselves into other people’s shoes yield higher task-dependent activations in this area (e.g., while doing a mentalizing task; Lamm, Meltzoff, & Decety, 2009; Masten et al., 2011). We suggest that baseline activation in the DMPFC might impact an individual’s general propensity to help strangers, possibly due to an increased capacity for social cognition that allows for a better understanding of strangers’ needs. This interpretation is also in line with the present questionnaire findings, which revealed a trend for a positive correlation between the perspective-taking scale and daily stranger-helping behavior (see Online Supplementary Analyses 1). Additionally, the observation that helping strangers is most often of a spontaneous nature (Amato, 1990) fits with the distinct neural signatures observed for friend-helping and stranger-helping behavior. That is, it is reasonable to assume that putting yourself into another person’s shoes is more relevant in situations of spontaneously helping a stranger compared to helping a friend in a planned manner. The present findings can also be understood within the framework of the empathy-altruism theory (e.g., Batson, Ahmad, Lishner, & Tsang, 2016; Batson & Shaw, 1991), which states that people are more likely to be helpful towards others when they feel empathic towards that person regardless of what they may receive in return. This mechanism of helping behavior is naturally most relevant when people help strangers, whom they may not meet again.

Several recent task-dependent and task-independent studies revealed a relationship between altruistic and generous choices and right temporoparietal junction (TPJ) (e.g., Gianotti et al., 2018; Krall et al., 2015; Morishima, Schunk, Bruhin, Ruff, & Fehr, 2012; Park et al., 2017; Tusche et al., 2016). Furthermore, a recent study by Strombach et al. (2015) found that the right TPJ showed increasing activation during generous choices as social distance increased between the participant and their playing partner. Our task-independent neural trait study did not reveal an effect in the right TPJ, neither for helping strangers nor for helping friends. However, helping strangers was associated with the DMPFC, a brain area strongly linked to the TPJ, both functionally and anatomically (e.g., Andrews-Hanna, Reidler, Sepulcre, Poulin, & Buckner, 2010; Baumgartner, Nash, Hill, & Knoch, 2015; Li, Mai, & Liu, 2014), and thought to be part of the same social network that plays a critical role in social cognition (e.g., Frith & Frith, 2012; Steinbeis, 2016; Van Overwalle, 2009). Although the exact roles these two brain region play in social cognition are still debated, it has been speculated that the TPJ is involved in more temporary and concrete social cognitions (e.g., inferring intentions and goals), whereas the DMPFC integrates (via connections to various brain regions, including the TPJ) social information across time and allows reflection and representation at a more abstract and complex cognitive level (e.g., Martin, Dzafic, Ramdave, & Meinzer, 2017; Van Overwalle, 2009). Thus, maybe our study on helping behavior in the daily life of participants triggered these more complex, high-order social cognitive processes in the DMPFC, whereas the other studies on generous and altruistic choices conducted in the laboratory might have triggered more basic, temporary social cognitive processes in the TPJ. Notably, we want to stress that these are speculative considerations and future studies are required to examine whether this distinction holds, and if it holds, what might be causing it.

Helping friends was negatively associated with slow-wave oscillations in the delta band, whereas helping strangers was positively associated with fast-wave oscillations in the beta bands. We interpreted the meaning of these bands in terms of cortical activation and cortical deactivation, which is supported by the literature (e.g., Laufs et al., 2003; Oakes et al., 2004). However, we acknowledge that some caution is warranted here as this interpretation might be a simplification, i.e., it might be meaningful that helping friends and helping strangers is associated with different frequency bands (besides the regional specificity). Since this is the first study showing this dissociation in the frequency bands in helping behavior, we believe that it is too early to speculate about it. This might be an interesting topic to pursue in future studies on helping behavior.

A potential limitation of our study is the use of self-report measures. Even though experience sampling overcomes some of the problems of traditional questionnaires (e.g., memory effects; Huber, Hill, & Lenz, 2012; Smallwood & O’Connor, 2011), we still rely on our subjects’ willingness to accurately report their behavior (Bolger, Davis, & Rafaeli, 2003). Thus, future studies might want to use other measures of helping behavior (costly helping paradigms in the behavioral laboratory, observation of real behavior in the field) to corroborate our findings.

Finally, we would like to note that the overall variance explained by the neural data is relatively small. Thus, the interpretations and conclusions should be treated with the necessary caution.

Taken together, the present findings offer potential psychological and cognitive mechanisms underlying an individual’s tendency to more frequently help friends or strangers. Namely, helping friends might be facilitated by a heightened capacity for self-control and strategic social behavior. Helping strangers, on the other hand, may be facilitated by a heightened capacity for social cognition, such as mentalizing and perspective-taking. More generally spoken, the present findings support what has been put forward recently, namely that social neuroscience could gain important new insights when considering the relational status between the giver and the recipient of prosocial behavior (Clark-Polner & Clark, 2014; Wlodarski & Dunbar, 2016).

## Electronic supplementary material

ESM 1 (DOCX 209 kb)
