# Supplementary material for: Frequency of helping friends and helping strangers is explained by different neural signatures
Source: Cogn Affect Behav Neurosci. 2018 Nov 7;19(1):177–86. doi: 10.3758/s13415-018-00655-2 (PMC6344399; doi:10.3758/s13415-018-00655-2)
Supplement: Supplementary file 1 — (DOCX 209 kb) [file 13415_2018_655_MOESM1_ESM.docx]

**Supplementary online material for**

# Frequency of helping friends and helping strangers is explained by different neural signatures

Anne Saulin^1*^, Thomas Baumgartner^1*^, Lorena R. R. Gianotti^1^, Wilhelm Hofmann^2^,

Daria Knoch^1^

^1^University of Bern, Institute of Psychology, Department of Social Psychology and Social Neuroscience, Switzerland

^2^University of Cologne, Social Cognition Center Cologne, Germany

*equal contribution

**Supplementary list of items used in the daily helping survey.**

For each item in the daily helping survey, participants had to indicate whether they had shown this behavior since the last time they filled out the survey and who the recipient of the behavior was (friend vs. stranger).

1. I held the door for someone.
2. I let someone skip the queue in the supermarket.
3. I gave someone positive feedback (unasked).
4. I offered someone my seat (e.g., on the bus).
5. I paid someone a favor.
6. I sacrificed time for someone (no matter if it was 5 minutes or a couple of hours).
7. I held the door of the tram or bus so someone could still catch it.
8. I picked something up from the floor for someone.
9. I helped someone with university work.
10. I asked someone whether he or she needed help.
11. I was there for someone who needed emotional or physical support.
12. I stood up for someone who was badly treated by others.

**
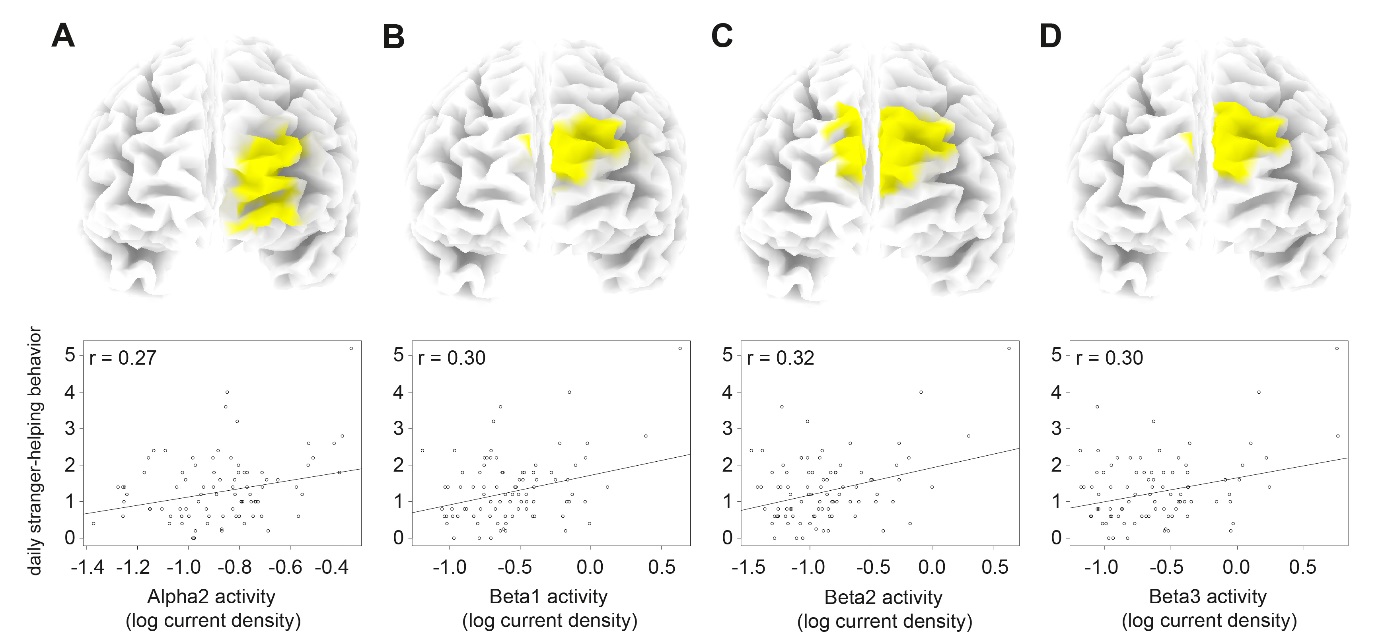
**

**Supplementary Fig. 1: Correlations between neural baseline activation and daily stranger-helping behavior**

Correlations between mean daily stranger-helping behavior and baseline activation (current density) in the dorsomedial prefrontal cortex in the alpha2 (column A), the beta1 (column B), the beta2 (column C), and the beta3 (column D) frequency bands. The upper panel visualizes the respective effect with the yellow shaded area designating those voxels passing the p < 0.05 threshold (whole-brain corrected). The lower panel comprises the corresponding scatterplots showing the robust regression lines, which account for potential outliers. The scatterplots are based on the extracted values from 10 mm spheres around the corresponding MNI peak coordinates. The pattern observed here is highly consistent across the beta bands as well as the alpha2 band. Since the alpha2 band is on the verge from the slowly oscillating bands to the fast ones and shows the same pattern as all three beta bands, we are confident in interpreting the findings in the alpha2 band in accordance with the findings in the beta bands. That is, oscillation in the alpha2 and beta bands likely reflect increased cortical activations.

**Supplementary Analyses 1: Correlations between personality questionnaires and helping behavior.**

The compliant subscale of the prosocial tendencies measure (PTM) correlated with daily friend- helping behavior (r = 0.27, n = 84, p < 0.05), and with daily stranger-helping behavior (r = 0.27, n = 84, p < 0.01). The dire subscale of the PTM displayed a trend correlation with daily friend- helping behavior (r = 0.21, n = 84, p < 0.1). Further, the anonymous subscale of the PTM showed a negative correlation with daily friend-helping behavior (r = -0.18, n = 84, p = 0.01). The other scales did not reveal significant correlations with daily friend-helping or stranger- helping behavior (all ps > 0.37). For the perspective-taking subscale of the interpersonal reactivity index (IRI), a trend correlation with daily stranger-helping behavior emerged (r = 0.19, n = 84, p < 0.1). No other associations between the IRI subscales and daily helping behavior reached significance or trend level (all ps > 0.14).

**Supplementary Analyses 2:**

Resting EEG has been shown to be highly stable, as outlined in the introduction of the manuscript. Here we examined whether we can provide evidence that the resting EEG is indeed stable in our sample. For that purpose, we tested whether the spectral analysis of the first 20 epochs (2-second epochs) of the eyes-closed condition is similar to the spectral analysis of the last 20 epochs (2-second epochs) of the eyes-closed condition. Note that on average our subjects had 88 epochs of eyes-closed resting state data. We focused in our analyses on the main regions and frequency bands reported in the analyses in the manuscript (correlation of friend-helping behavior with current density in the right DLPFC in the delta band and correlation of stranger-helping behavior with current density in the DMPFC in the three beta bands and in the alpha2 band). As expected (based on the consistent findings reported in the literature), we found strong evidence that resting EEG is highly stable in our sample. Current density in the first 20 epochs were highly correlated with current density in the last 20 epochs in all regions and frequency bands of our main findings (all r > 0.872). Please see the following table for details:

| Brain region | Band | r-value | p-value |
| --- | --- | --- | --- |
| DLPFC (right) | Delta | 0.914 | p < 0.0001 |
| DMPFC | Alpha 2 | 0.872 | p < 0.0001 |
| DMPFC | Beta 1 | 0.936 | p < 0.0001 |
| DMPFC | Beta 2 | 0.914 | p < 0.0001 |
| DMPFC | Beta 3 | 0.929 | p < 0.0001 |

**Supplementary Analyses 3:**

In order to strengthen our exploratory findings, we attempted an internal replication of your main findings in the DLPFC and DMPFC. For that purpose, we examined whether we find a similar predictive pattern in the DLPFC and DMPFC in the eyes-open condition as we have demonstrated for the eyes-closed condition in the results section of the manuscript. Interestingly, the findings indeed revealed a highly similar predictive pattern in the DLPFC and DMPFC in the eyes-open condition as in the eyes-closed condition. More precisely, robust regression using the resting EEG of the eyes-open condition revealed that current density in the DLPFC in the delta band showed a highly similar (as in the eyes-closed condition) negative correlation with daily friend-helping behavior (r = -0.351, p = 0.001). Furthermore, robust regressions using the resting EEG of the eyes-open condition revealed that current density in the three beta bands and the alpha2 band also showed highly similar (as in the eyes-closed condition) positive correlations with daily stranger-helping behavior (beta1: r = 0.353, p = 0.001, beta2: r = 0.323, p = 0.002, beta3: r = 0.294, p = 0.005, alpha2: r = 0.289, p = 0.006). Furthermore, Meng’s test for dependent correlations provided statistical evidence that the correlations for the eyes-closed and eyes-open condition are not significantly different (Delta band: z = -0.228, p = 0.820, beta1: z = -0.666, p = 0.512, beta2: z = 0.127, p = 0.898, beta3: z = 0.272, p = 0.786, alpha2: z = -0.114, p = 0.910). Thus, these results demonstrates that our findings hold, irrespective of whether we analyse the eyes-closed or eyes-open condition.

**Supplementary Analyses 4:**

As reported in the manuscript, the time delay between the acquisition of the resting EEG and the experience sampling showed some variability (several weeks). In order to examine whether this variability has an impact on the results reported in the main manuscript, we used this variability as an additional covariate in our analyses. Notably, all our findings hold, i.e. we find highly similar results when we control for the time delay in our analyses. More precisely, robust regression (including the covariate time delay) revealed a highly similar negative correlation between daily friend-helping behavior and current density in the right DLPFC in the delta frequency band (r = -0.31, p = 0.001). Moreover, robust regression (including the covariate time delay) also revealed highly similar positive correlations between daily stranger-helping behavior and current density in the DMPFC in the beta1 (r = -0.29, p = 0.002), beta2 (r = -0.31, p = 0.001), beta3 (r = -0.30, p = 0.001) and alpha2 (r = -0.26, p = 0.005) frequency bands. Thus, these findings clearly indicate that the time delay between the acquisitions of our measurements has no impact on the reported predictive pattern in the DLPFC and DMPFC.

**Supplementary Table 1**

Distribution of the questionnaire data

| Scale | Mean | SD | 25 % quartile | 75 % quartile |
| --- | --- | --- | --- | --- |
| PTM - public | 1.47 | 0.53 | 1.00 | 1.75 |
| PTM - anonymous | 2.38 | 0.92 | 1.60 | 3.00 |
| PTM - dire | 3.22 | 0.87 | 2.67 | 4.00 |
| PTM - emotional | 3.07 | 0.89 | 2.50 | 3.75 |
| PTM - compliant | 4.16 | 0.69 | 4.00 | 5.00 |
| PTM - altruism | 1.61 | 0.44 | 1.40 | 1.80 |
| IRI – perspective taking | 3.27 | 0.26 | 3.00 | 3.43 |
| IRI - empathy | 3.25 | 0.40 | 3.00 | 3.43 |

**Supplementary Table 2**

Comparison of robust regression models. Model 1 includes the questionnaire data and Model 2 includes the same predictors as Model 1 with the addition of the brain data. For details on the coefficients, please see supplementary Tables 3 and 4.

| **Dependent variable** | **models** | **R²** | **ΔR²** | **Wald test**  **Test statistics** | **Wald test**  **p-value** |
| --- | --- | --- | --- | --- | --- |
| Daily friend helping | Model 1^a^ | .151 |  |  |  |
|  | Model 2^b^ | .242 | .091 | 5.77 | .016* |
| Daily stranger helping | Model 1^a^ | .147 |  |  |  |
|  | Model 2^c^ | .268 | .121 | 10.85 | .001** |

a. predictors: (intercept), PTM-public, PTM-anonymous, PTM-dire, PTM-emotional, PTM-compliant, PTM altruism, IRI-perspective taking, IRI-empathy

b. predictors: (intercept), PTM-public, PTM-anonymous, PTM-dire, PTM-emotional, PTM-compliant, PTM altruism, IRI-perspective taking, IRI-empathy, baseline activation in the DLPFC in the delta band

c. predictors: (intercept), PTM-public, PTM-anonymous, PTM-dire, PTM-emotional, PTM-compliant, PTM altruism, IRI-perspective taking, IRI-empathy, baseline activation in the DMPFC in the beta2 band

**Supplementary Table 3**

Coefficients for models predicting daily friend-helping behavior. Model 1 comprises the questionnaire data as predictors. Model 2 comprises the same predictors as Model 1 with the addition of the neural baseline activation in DLPFC in the delta band. For model comparison, please see supplementary Table 2.

|  | **predictors** | **Unstandardized coefficients**    **Betas Std. error** | | **Standardized**  **Coefficients**  **Betas** | **t-value** | **p-value** |
| --- | --- | --- | --- | --- | --- | --- |
| Model 1 | (Intercept) | 0.662 | 7.008 | -0.047 | 0.090 | 0.925 |
|  | PTM - public | 0.013 | 0.866 | 0.002 | 0.010 | 0.988 |
|  | PTM - anonymous | -0.875 | 0.467 | -0.215 | -1.870 | 0.065 |
|  | PTM - dire | 0.406 | 0.700 | 0.094 | 0.580 | 0.564 |
|  | PTM - emotional | 0.157 | 0.843 | 0.037 | 0.190 | 0.852 |
|  | PTM - compliant | 1.227 | 0.718 | 0.227 | 1.710 | 0.091 |
|  | PTM - altruism | -1.026 | 1.162 | -0.119 | -0.880 | 0.380 |
|  | IRI – perspective taking | 0.145 | 1.788 | 0.010 | 0.080 | 0.936 |
|  | IRI - empathy | 0.856 | 1.260 | 0.092 | 0.680 | 0.499 |
| Model 2 | (Intercept) | 3.099 | 6.493 | -0.064 | 0.480 | 0.635 |
|  | PTM - public | -0.414 | 0.813 | -0.059 | -0.510 | 0.612 |
|  | PTM - anonymous | -0.847 | 0.431 | -0.208 | -1.960 | 0.053 |
|  | PTM - dire | 0.289 | 0.649 | 0.067 | 0.440 | 0.658 |
|  | PTM - emotional | 0.648 | 0.794 | 0.154 | 0.820 | 0.417 |
|  | PTM - compliant | 0.817 | 0.671 | 0.151 | 1.220 | 0.227 |
|  | PTM - altruism | -0.655 | 1.073 | -0.076 | -0.610 | 0.543 |
|  | IRI – perspective taking | 0.196 | 1.643 | 0.014 | 0.120 | 0.905 |
|  | IRI - empathy | 0.015 | 1.196 | 0.002 | 0.010 | 0.990 |
|  | DLPFC delta | -4.617 | 1.627 | -0.308 | -2.840 | 0.006** |

**Supplementary Table 4**

Coefficients for models predicting daily stranger-helping behavior. Model 1 comprises the questionnaire data as predictors. Model 2 comprises the same predictors as Model 1 with the addition of the neural baseline activation in DMPFC in the beta2 band. For model comparison, please see supplementary Table 2.

|  | **predictors** | **Unstandardized coefficients**    **Betas Std. error** | | **Standardized**  **Coefficients**  **Betas** | **t-value** | **p-value** |
| --- | --- | --- | --- | --- | --- | --- |
| Model 1 | (Intercept) | 0.321 | 1.463 | -0.072 | 0.220 | 0.827 |
|  | PTM - public | 0.160 | 0.178 | 0.094 | 0.900 | 0.370 |
|  | PTM - anonymous | 0.067 | 0.095 | 0.069 | 0.710 | 0.482 |
|  | PTM - dire | -0.117 | 0.144 | -0.112 | -0.810 | 0.418 |
|  | PTM - emotional | 0.069 | 0.175 | 0.068 | 0.400 | 0.694 |
|  | PTM - compliant | 0.297 | 0.148 | 0.228 | 2.000 | 0.049* |
|  | PTM - altruism | -0.466 | 0.244 | -0.224 | -1.910 | 0.060 |
|  | IRI – perspective taking | 0.198 | 0.368 | 0.057 | 0.540 | 0.592 |
|  | IRI - empathy | -0.126 | 0.263 | -0.056 | -0.480 | 0.633 |
| Model 2 | (Intercept) | 0.926 | 1.405 | -0.049 | 0.660 | 0.512 |
|  | PTM - public | 0.189 | 0.169 | 0.112 | 1.120 | 0.265 |
|  | PTM - anonymous | 0.062 | 0.090 | 0.063 | 0.690 | 0.492 |
|  | PTM - dire | -0.128 | 0.138 | -0.123 | -0.930 | 0.356 |
|  | PTM - emotional | 0.084 | 0.167 | 0.083 | 0.500 | 0.617 |
|  | PTM - compliant | 0.321 | 0.140 | 0.246 | 2.290 | 0.025* |
|  | PTM - altruism | -0.567 | 0.233 | -0.272 | -2.430 | 0.018 |
|  | IRI – perspective taking | 0.177 | 0.348 | 0.051 | 0.510 | 0.613 |
|  | IRI - empathy | -0.082 | 0.249 | -0.036 | -0.330 | 0.743 |
|  | DMPFC beta2 | 0.724 | 0.223 | 0.313 | 3.240 | 0.002** |

**Supplementary Table 5**

Comparison of robust regression models. Model 1 includes the questionnaire and demographic (age and gender) data and Model 2 includes the same predictors as Model 1 with the addition of the brain data. For details on the coefficients, please see supplementary Tables 6 and 7.

| **Dependent variable** | **models** | **R²** | **ΔR²** | **Wald test**  **Test statistics** | **Wald test**  **p-value** |
| --- | --- | --- | --- | --- | --- |
| Daily friend helping | Model 1^a^ | .217 |  |  |  |
|  | Model 2^b^ | .318 | .101 | 5.39 | .020* |
| Daily stranger helping | Model 1^a^ | .164 |  |  |  |
|  | Model 2^c^ | .272 | .108 | 9.94 | .002** |

a. predictors: (intercept), age, gender, PTM-public, PTM-anonymous, PTM-dire, PTM-emotional, PTM-compliant, PTM altruism, IRI-perspective taking, IRI-empathy

b. predictors: (intercept), age, gender, PTM-public, PTM-anonymous, PTM-dire, PTM-emotional, PTM-compliant, PTM altruism, IRI-perspective taking, IRI-empathy, baseline activation in the DLPFC in the delta band

c. predictors: (intercept), age, gender, PTM-public, PTM-anonymous, PTM-dire, PTM-emotional, PTM-compliant, PTM altruism, IRI-perspective taking, IRI-empathy, baseline activation in the DMPFC in the beta2 band

**Supplementary Table 6**

Coefficients for models predicting daily friend-helping behavior. Model 1 comprises the predictors age, gender, and questionnaire data. Model 2 comprises the same predictors as Model 1 with the addition of the neural baseline activation in DLPFC in the delta band. For model comparison, please see supplementary Table 5.

|  | **predictors** | **Unstandardized coefficients**    **Betas Std. error** | | **Standardized**  **Coefficients**  **Betas** | **t-value** | **p-value** |
| --- | --- | --- | --- | --- | --- | --- |
| Model 1 | (Intercept) | 10.959 | 9.070 | -0.060 | 1.210 | 0.231 |
|  | gender | -2.035 | 1.097 | -0.229 | -1.850 | 0.068 |
|  | age | -0.344 | 0.214 | -0.196 | -1.610 | 0.113 |
|  | PTM - public | -0.185 | 0.879 | -0.026 | -0.210 | 0.834 |
|  | PTM - anonymous | -0.599 | 0.483 | -0.147 | -1.240 | 0.219 |
|  | PTM - dire | 0.220 | 0.708 | 0.051 | 0.310 | 0.757 |
|  | PTM - emotional | 0.008 | 0.842 | 0.002 | 0.010 | 0.993 |
|  | PTM - compliant | 1.318 | 0.718 | 0.244 | 1.840 | 0.071 |
|  | PTM - altruism | -1.133 | 1.171 | -0.131 | -0.970 | 0.336 |
|  | IRI – perspective taking | 0.123 | 1.788 | 0.009 | 0.070 | 0.945 |
|  | IRI - empathy | 1.208 | 1.306 | 0.130 | 0.930 | 0.358 |
| Model 2 | (Intercept) | 13.288 | 8.894 | -0.061 | 1.490 | 0.140 |
|  | gender | -2.160 | 1.086 | -0.243 | -1.990 | 0.051 |
|  | age | -0.304 | 0.212 | -0.173 | -1.430 | 0.156 |
|  | PTM - public | -0.708 | 0.884 | -0.101 | -0.800 | 0.426 |
|  | PTM - anonymous | -0.610 | 0.475 | -0.150 | -1.280 | 0.203 |
|  | PTM - dire | 0.070 | 0.693 | 0.016 | 0.100 | 0.920 |
|  | PTM - emotional | 0.550 | 0.847 | 0.131 | 0.650 | 0.518 |
|  | PTM - compliant | 0.975 | 0.724 | 0.180 | 1.350 | 0.182 |
|  | PTM - altruism | -0.737 | 1.153 | -0.085 | -0.640 | 0.525 |
|  | IRI – perspective taking | -0.115 | 1.752 | -0.008 | -0.070 | 0.948 |
|  | IRI - empathy | 0.477 | 1.320 | 0.051 | 0.360 | 0.719 |
|  | DLPFC delta | -4.470 | 1.745 | -0.298 | -2.560 | 0.013* |

**Supplementary Table 7**

Coefficients for models predicting daily stranger-helping behavior. Model 1 comprises the predictors age, gender, and questionnaire data. Model 2 comprises the same predictors as Model 1 with the addition of the neural baseline activation in DMPFC in the beta2 band. For model comparison, please see supplementary Table 5.

|  | **predictors** | **Unstandardized coefficients**    **Betas Std. error** | | **Standardized**  **Coefficients**  **Betas** | **t-value** | **p-value** |
| --- | --- | --- | --- | --- | --- | --- |
| Model 1 | (Intercept) | 1.138 | 1.802 | -0.064 | 0.630 | 0.530 |
|  | gender | 0.104 | 0.218 | 0.049 | 0.480 | 0.635 |
|  | age | -0.039 | 0.043 | -0.093 | -0.920 | 0.361 |
|  | PTM - public | 0.182 | 0.174 | 0.107 | 1.040 | 0.300 |
|  | PTM - anonymous | 0.082 | 0.095 | 0.083 | 0.860 | 0.395 |
|  | PTM - dire | -0.153 | 0.141 | -0.147 | -1.090 | 0.280 |
|  | PTM - emotional | 0.077 | 0.169 | 0.076 | 0.460 | 0.648 |
|  | PTM - compliant | 0.335 | 0.145 | 0.256 | 2.310 | 0.024* |
|  | PTM - altruism | -0.510 | 0.239 | -0.245 | -2.130 | 0.036* |
|  | IRI – perspective taking | 0.174 | 0.357 | 0.050 | 0.490 | 0.627 |
|  | IRI - empathy | -0.168 | 0.259 | -0.075 | -0.650 | 0.519 |
| Model 2 | (Intercept) | 1.205 | 1.835 | -0.053 | 0.660 | 0.513 |
|  | gender | 0.140 | 0.223 | 0.066 | 0.630 | 0.531 |
|  | age | -0.020 | 0.044 | -0.047 | -0.460 | 0.648 |
|  | PTM - public | 0.200 | 0.178 | 0.118 | 1.120 | 0.265 |
|  | PTM - anonymous | 0.066 | 0.096 | 0.067 | 0.690 | 0.495 |
|  | PTM - dire | -0.154 | 0.146 | -0.147 | -1.060 | 0.295 |
|  | PTM - emotional | 0.095 | 0.174 | 0.094 | 0.550 | 0.587 |
|  | PTM - compliant | 0.328 | 0.147 | 0.251 | 2.240 | 0.028* |
|  | PTM - altruism | -0.583 | 0.245 | -0.280 | -2.380 | 0.020* |
|  | IRI – perspective taking | 0.187 | 0.362 | 0.054 | 0.520 | 0.608 |
|  | IRI - empathy | -0.126 | 0.263 | -0.056 | -0.480 | 0.634 |
|  | DMPFC beta2 | 0.693 | 0.234 | 0.300 | 2.960 | 0.004** |
